# PatchDSA: improving digital subtraction angiography with patch-based phase-matching in natural breathing scenarios

**DOI:** 10.1007/s12194-025-00922-1

**Published:** 2025-06-10

**Authors:** Yuki Sekiguchi, Takayuki Okamoto, Tsukiho Matsuzawa, Kentaro Fujimoto, Kisako Fujiwara, Takayuki Kondo, Jun Koizumi, Hideaki Haneishi

**Affiliations:** 1 Graduate School of Science and Engineering, Chiba University, Chiba, Japan; 2 Center for Frontier Medical Engineering, Chiba University, Chiba, Japan; 3 Faculty of Engineering, Chiba University, Chiba, Japan; 4 Department of Gastroenterology, Graduate School of Medicine, Chiba University, Chiba, Japan; 5 Department of Comprehensive Radiology, Graduate School of Medicine, Chiba University, Chiba, Japan

**Keywords:** Abdominal angiogram, Digital subtraction angiography (DSA), Phase-matching, Natural breathing, X-ray fluoroscopy

## Abstract

Digital subtraction angiography (DSA) is used to visualize blood vessels by subtracting pre-contrast (mask) images from contrast images; sequential mask and contrast images are used to generate dynamic DSA images that allow observation of blood flow and organ movements. However, misalignment between mask and contrast images can cause motion artifacts, which not only obscure the appearance of enhanced structures but also lead to the misidentification of patterns as vascular structures. In this study, we proposed a new method for generating abdominal sequential DSA images using a patch-based phase-matching technique between mask and contrast images acquired under natural breathing conditions. Our method divides mask and contrast images into small patches and selects the mask image patch most structurally similar to each patch in the target contrast image. Furthermore, the selected mask image patch is refined by searching for the subpixel-level region that most closely matches the target contrast image patch. The proposed method was evaluated using 20 abdominal angiogram cases, and its performance was compared with an existing phase matching–based method. Our experimental results showed that the proposed method effectively reduced motion artifacts and outperformed the comparison method in all cases. We demonstrated that our method successfully identified the optimal mask image for each contrast image on a patch-by-patch basis, allowing it to suppress artifacts caused by physiological motions such as peristalsis and cardiac pulsation, thereby generating higher-quality DSA images.

## Introduction

X-ray fluoroscopy is a medical imaging technique that provides dynamic, real-time views of internal body structures. It is an excellent tool for observing organs and tissues in motion and for guiding medical instruments during diagnostic and therapeutic procedures, such as catheter insertion and stent placement. In interventional radiology, real-time fluoroscopic imaging plays a crucial role in monitoring the target vasculature, tracking organ deformations, and accurately pinpointing the location of medical instruments, thereby ensuring the safety and precision of minimally invasive treatments. Additionally, the visibility of blood vessels on fluoroscopic images can be enhanced by injecting contrast agents via a catheter, thus providing more dynamic and clearer views of vascular structures. However, blood vessels often overlap with themselves and surrounding organs in fluoroscopic imaging, making it difficult to distinguish between them.

Digital subtraction angiography (DSA) [1–3] is a specialized fluoroscopic technique primarily designed for detailed visualization of blood vessels. By employing a straightforward postprocessing algorithm that subtracts pre-contrast images, called mask images, from contrast images [4], DSA effectively eliminates background structures from images and enhances the clarity of the contrast materials within blood vessels. Furthermore, sequential mask and contrast images can be used to generate dynamic DSA images that enable the observation of blood flow and the dynamic movement of organs. This capability is especially important for abdominal DSA. Unlike cerebral DSA, where organ motion and deformation are relatively minimal, abdominal DSA is affected by significant organ motion and deformation due to cardiovascular and respiratory movements, which frequently obscure the target vasculature and make accurate observation using two-dimensional (2D) images alone difficult. These challenges underscore the importance of incorporating time-resolved imaging to more precisely capture the movement of flowing blood in overlapping vessels and to detect culprit arteries. For example, in interventional procedures such as transarterial embolization (TAE) for the treatment of conditions such as hepatocellular carcinoma or traumatic injuries, detailed analysis of blood flow using dynamic observation—including monitoring the direction of blood flow and identifying feeding or bleeding points by reviewing DSA frames—enhances both procedural accuracy and therapeutic outcomes.

**Fig. 1:**
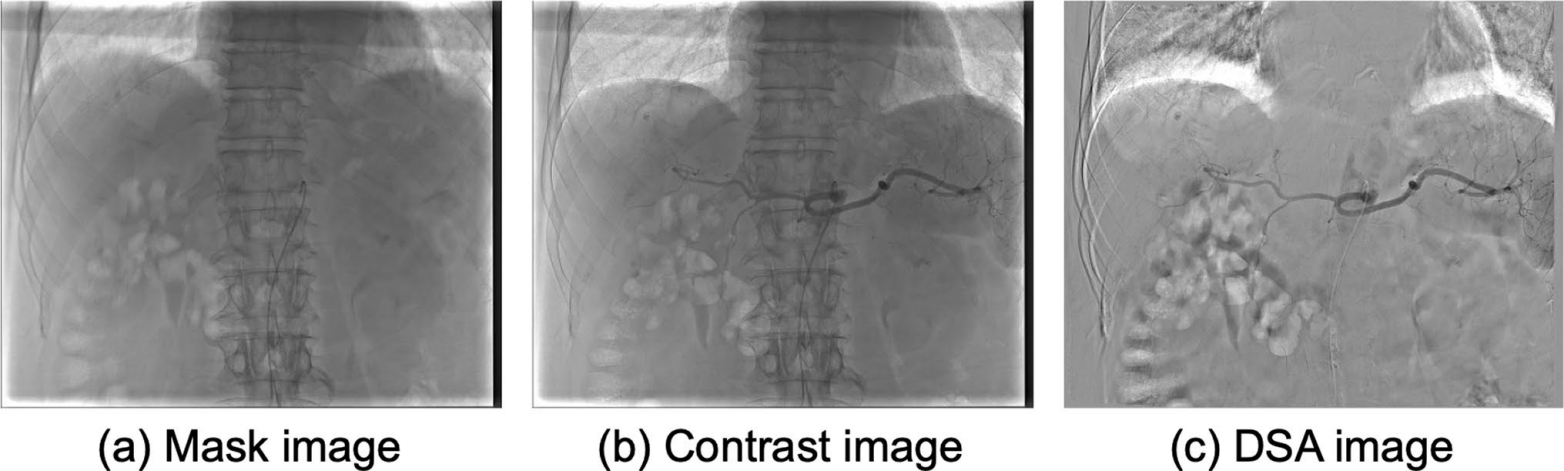
Example of motion artifacts in an abdominal DSA image. **a** Mask image, **b** contrast image, and **c** DSA image generated by subtracting (**a**) from (**b**)

A major drawback of DSA is the occurrence of motion artifacts caused by the misalignment of mask and contrast images [5, 6]. An example of a failed abdominal DSA image in which motion artifacts are visible is shown in Fig. 1(c). These severe motion artifacts not only obscure the appearance of enhanced vascular structures but also result in patterns that can be incorrectly identified as vascular structures. Furthermore, inadequate visualization of vascular structures often necessitates reacquisition, which requires additional contrast injections and results in increased radiation exposure and time [4, 7].

To obtain clear abdominal DSA images, patients are requested to control their breathing when necessary, such as by temporarily holding their breath during acquisitions. However, these requirements can pose a significant challenge or burden for patients with ascites, elderly patients, and those who have difficulty controlling their breathing [8, 9]. Therefore, an approach that enables the acquisition of sequential DSA images under natural breathing conditions is needed.

Ohnishi et al*.* [10] proposed a respiratory phase–matching method for generating clear sequential DSA images using preoperative contrast images and intra-operative mask images collected under natural breathing. Using a pattern-matching technique, their method selects contrast image exhibiting the most similar respiratory phase for each mask image and then generates subtraction images. Optimal matching between contrast images and mask images allows for the generation of acceptable DSA images without the need for breath-holding. However, the respiratory phase–matching method proposed by Ohnishi et al. relies on evaluating of the similarity between two images based on a region of interest placed near the diaphragm. This approach does not fully account for the complex motion patterns of other abdominal organs that are not directly related to movement of the diaphragm, such as intestinal peristalsis and cardiovascular pulsatility, which can lead to potential mismatches and inaccuracies [8, 9].

In this study, we propose a new method for abdominal sequential DSA generation that employs patch-based phase-matching between mask and contrast images acquired under natural breathing conditions. In the proposed method, both the mask and contrast images are divided into small patches. For each patch in the target contrast image, the most structurally similar patch is selected from a series of mask image patches. The selected mask image patch is then further refined by searching for the area that most closely matches the target contrast image patch at a subpixel level. We hypothesize that identifying the optimal mask image for each contrast image on a patch-by-patch basis, instead of relying on the entire image, can more effectively suppress artifacts caused by physiological motions such as peristalsis and cardiac pulsation, thereby generating higher-quality DSA images than conventional methods. We demonstrate the effectiveness of the proposed method compared with the method previously developed by Ohnishi et al*.* [10] (Ohnishi’s method) through qualitative and quantitative evaluations using 20 abdominal angiogram cases.

### Related works

To date, various methods have been proposed to generate DSA images with reduced motion artifacts, which can be broadly categorized into registration-based and direct generation methods.

Registration-based methods [11–23] focus on aligning the mask and contrast images using image registration strategies. For example, Bentoutou et al. [12] proposed an automatic control point extraction method for the contrast image and introduced a technique for identifying corresponding points in the mask image. Their method employs affine transformation and thin-plate spline [24] interpolating registration to achieve precise alignment. Nejati et al*.* [16] proposed an elastic registration algorithm based on a multiresolution search strategy that iteratively decomposes both the mask and the contrast images from coarse to fine levels to align the two images. Although these methods effectively reduce motion artifacts, registration techniques can cause excessive image deformation that could distort the appearance of blood vessels and organs. Moreover, these methods are computationally expensive, with the processing burden increasing when applied to multiple images.

By contrast, Su et al*.* [23] proposed a deep learning (DL)-based motion correction framework known as AngioMoCo, which was designed to correct for motion in cerebral angiographic images and based on the VoxelMorph framework [25]. AngioMoCo was shown to outperform both iterative affine registration and other DL-based techniques and can process an entire series in less than 1 s on GPUs. However, compared with cerebral angiograms, abdominal angiograms exhibit more dynamic motion, which poses a risk of insufficient artifact reduction and thus the introduction of undesirable artifacts.

Direct generation methods [7, 8, 26–28] eliminate the need for traditional subtraction using a mask image. Instead, these methods directly generate subtraction images based solely on contrast images. Ueda et al. [7] developed a DL-based angiogram translation model inspired by the pix2pix network [29]. Their model extracts contrast-enhanced areas from dynamic angiograms, thus enabling the generation of DSA-like cerebral angiograms. Yonezawa et al. [8] used a similar approach to generate DSA-like abdominal angiograms. As these methods demonstrate the capability of generating DSA images with fewer artifacts, they could be applied under natural breathing conditions. However, while these methods can enhance the contrast of larger blood vessels, improving the contrast of finer vessels is often challenging. Furthermore, because direct generation methods do not explicitly compensate for motion-induced misalignment between images, they may introduce inaccuracies, such as hallucinations or modification of contrast and vessels, thereby diminishing interpretability [23].

In this study, we adopted a straightforward and practical subtraction-based method that assumes the availability of multiple consecutive mask and contrast images acquired under natural breathing conditions, as commonly used in interventional treatments involving DSA. The proposed method identifies the optimal mask image patch for each contrast image patch and obtains the DSA image patch by computing the difference between the two patches. To the best of our knowledge, the proposed method represents a unique approach for generating DSA images.

## Materials and methods

### Datasets

Intraoperative abdominal angiograms collected using a digital X-ray imaging system (DFP-8000D, Canon Medical Systems Co., Tochigi, Japan) were retrospectively obtained for 20 cases at Chiba University Hospital between July 2022 and September 2023. The inclusion criteria required a series of mask images covering a single respiratory phase, followed by a subsequent series of contrast images, with both image sets acquired under natural breathing conditions. These cases included the following procedures: transarterial chemoembolization (TACE) in 12 cases, transarterial infusion therapy (TAI) in 2 cases, hepatic arterial infusion chemotherapy (HAIC) in 1 case, diagnostic angiography in 4 cases, and hepatic venous pressure measurement with contrast-enhanced angiography in 1 case. The dataset comprised 17 male and three female patients, with a mean age of 72.4 years. Both image sets consisted of approximately 30 frames per mask series and 50–70 frames per contrast series. The frame rate for both series was 6 frames per second. The matrix size was 512 × 512 pixels, and the pixel spacing varied across cases but was approximately 0.57 × 0.57 mm^2^. Additionally, unnecessary regions outside the fluoroscopic field were cropped from the acquired images, resulting in a final matrix size of approximately 370 × 490 pixels. The study protocol and determination of study participants were conducted in accordance with accepted ethical standards, following approval by the Ethical Review Board of Chiba University (approval number 5–491).

### DSA image generation

The goal of this study was to generate sequential DSA images using a series of mask and contrast images acquired under natural breathing conditions. Multiple individual DSA images were generated using the proposed method, and these were sequentially combined to produce a final time-series DSA sequence. The process of generating each DSA image comprises three steps: (A) patch-based phase-matching, (B) fine-tuning based on structural similarities, and (C) DSA image generation with brightness correction. A detailed description of each step is provided below.

#### A) Patch-based phase-matching

An intraoperative abdominal angiogram acquired under natural breathing conditions includes two types of images: a series of mask images and a series of contrast images. If the series of mask images is described by $\mathcal{M}={\left\{{M}_{t}\right\}}_{t=1}^{T}$, where ${M}_{t}\in {\mathbb{R}}^{H\times W}$ represents the *t*-th mask image and $T$ denotes the total number of mask images in the series, and $H$ and $W$ indicate the height and width of each image, respectively. Similarly, a given target image among the series of contrast images is described by $C\in {\mathbb{R}}^{H\times W}$. All images are divided into $N\times N$ smaller patches*,* resulting in the sets $\left\{{c}^{\text{1,1}},\cdots ,{c}^{i,j},\cdots ,{c}^{N,N}\right\}$ for the contrast image patches and ${\left\{\left\{{m}_{t}^{\text{1,1}},\cdots ,{m}_{t}^{i,j},\cdots ,{m}_{t}^{N,N}\right\}\right\}}_{t=1}^{T}$ for the mask image patches. Here, ${c}^{i,j}\in {\mathbb{R}}^{\frac{H}{N}\times \frac{W}{N}}$ and ${m}_{t}^{i,j}\in {\mathbb{R}}^{\frac{H}{N}\times \frac{W}{N}}$ represent the contrast and mask image patches, respectively. $N$ indicates the number of divisions along each axis, and $(i,j)$ denotes the grid location of a given patch. In this study, the number of patch divisions $N$ was empirically set to 10, as this value provided a practical balance: smaller patches often lacked sufficient structural information for robust matching, whereas larger patches tended to overlook local anatomical variations.

**Fig. 2:**
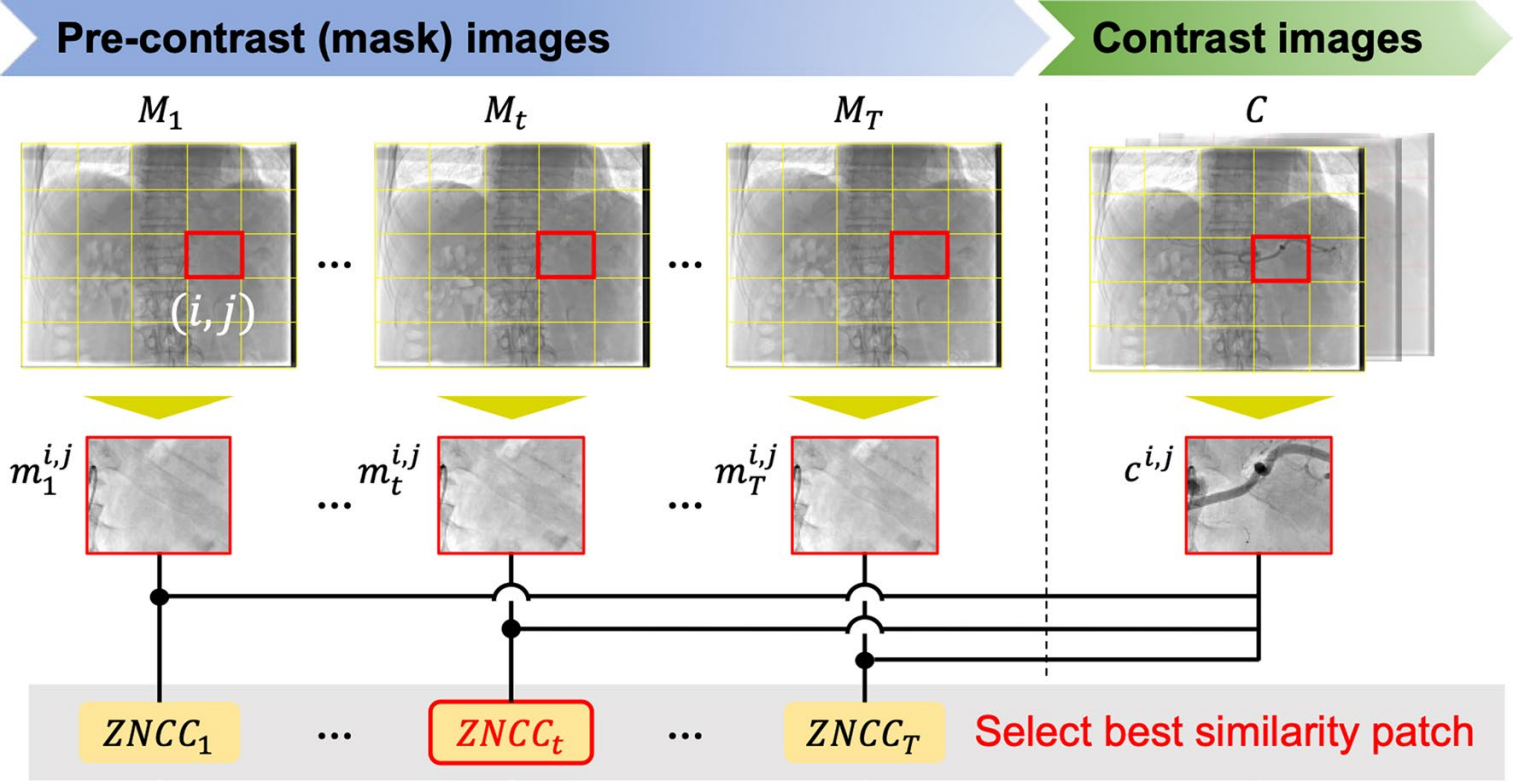
Overview of the patch-based phase-matching method

For each contrast image patch ${c}^{i,j}$, a pattern-matching technique was used to calculate the similarity with the corresponding mask image patch at the same location $\left\{{m}_{1}^{i,j},\cdots ,{m}_{t}^{i,j},\cdots ,{m}_{T}^{i,j}\right\}$ in order to select the most structurally similar mask patch. In this process, zero normalized cross-correlation (ZNCC) is employed as the metric to quantitatively evaluate the similarity between patches. The patch with the highest ZNCC value was selected as the optimal patch. Figure 2 illustrates the outline of the patch-based phase-matching process.

#### B) Fine-tuning based on structural similarities

In step A, we selected the mask image patch ${m}_{t}^{i,j}$, which is structurally similar to the contrast image patch ${c}^{i,j}$*.* However, some organs can shift in various directions unrelated to the movement of the diaphragm. To address this possibility, we fine-tuned the selection by incrementally shifting ${m}_{t}^{i,j}$ within the whole mask image ${M}_{t}$ at a subpixel level, locating the position that exhibited greater structural similarity.

In step B, we searched ${M}_{t}$ for a new mask image patch, denoted ${\widehat{m}}_{t}^{i,j}$, that would exhibit greater structural similarity to ${c}^{i,j}$ than the initially selected patch, ${m}_{t}^{i,j}$. The mask image patch was shifted within the 2D space of ${M}_{t}$, guided by the parallel translation parameters ${t}_{x}$ and ${t}_{y}$, starting from the initial position of ${m}_{t}^{i,j}$. ZNCC was also used to assess the degree of similarity between patches, and the patch with the highest ZNCC value was selected as the optimal patch. The optimal parallel translation parameters were determined using the downhill simplex method. The above process was repeated for each contrast image patch.

#### C) DSA image generation with brightness correction

Before performing patch-wise subtraction, brightness correction was applied to the selected mask image patch because brightness variations across mask image patches can cause tiling artifacts and lead to inconsistencies across the DSA image patches. Ensuring consistent brightness is important for generating a visually uniform DSA image. The correction process was designed to align the average pixel value of each mask image patch with that of its corresponding contrast image patch. This adjustment was performed using the following equation:

1 $${m}_{after}^{i,j}\left(x,y\right)={m}_{before}^{i,j}\left(x,y\right)+{\mu }_{c}^{i,j}-{\mu }_{m}^{i,j}$$

where ${m}_{before}^{i,j}\left(x,y\right)$ and ${m}_{after}^{i,j}\left(x,y\right)$ represent the pixel values at position $(x,y)$ in the mask image patch before and after brightness correction_**,**_ respectively. The terms ${\mu }_{m}^{i,j}$ and ${\mu }_{c}^{i,j}$ represent the mean values of the mask and contrast image patches_**,**_ respectively.

When calculating the mean value of the contrast image patch ${\mu }_{c}^{i,j}$, it is necessary to exclude the contrast-enhanced regions. These regions are unique to the contrast image and are not present in the mask image. Therefore, including them can lead to an underestimation of the mean value, resulting in excessive brightness correction. To address this issue, contrast-enhanced regions were excluded based on a pixel intensity threshold. The threshold value was determined by a researcher (YS), who specializes in medical image processing, using a preliminary DSA image patch—generated prior to applying brightness correction to the mask image patch—for identifying the contrast-enhanced regions.

The brightness correction step ensures that each mask image patch closely matches the brightness of its corresponding contrast image patch, promoting a seamless appearance when the DSA image patches are later integrated. After correction, DSA image patches were generated by subtracting the corrected mask image patches from the corresponding target contrast image patches and then combined to construct the complete DSA image.

### Experimental setup

#### Comparison method

We compared the performance of the proposed method with that of Ohnishi’s method [10]. To implement Ohnishi’s method, we modified the workflow to ensure a fair comparison of DSA image quality using our dataset. Specifically, instead of using the mask image as the target and searching for a contrast image with a similar respiratory phase—as originally done in Ohnishi’s method—we reversed the selection process. In our implementation, the contrast image was defined as the target, and the most phase-matched mask image was selected, following the same procedure as in the proposed method. This modification was necessary due to differences in the data acquisition workflow; however, the core algorithm and matching criteria remained unchanged. All methods were implemented in C++ using shared-memory parallel processing on an Intel Core i9-13900H CPU (2.60 GHz, 14 cores) with 32 GB of RAM.

#### Evaluation

The generated DSA images were evaluated both qualitatively and quantitatively. For qualitative evaluation, a physician (K. Fujimoto) with more than nine years of clinical experience visually reviewed the results of both Ohnishi’s method and the proposed method. The evaluator is board certified in gastroenterologist of the Japanese society of gastroenterology and in Hepatologist of the Japan Society of Hepatology. For quantitative evaluation, we calculated the entropy [30] for each DSA image, defined as follows:

2 $$Entropy=-\sum_{{g=g}_{\text{min}}}^{{g}_{\text{max}}}{p}_{g}\text{log}\left({p}_{g}\right),$$

where ${g}_{\text{min}}$ and ${g}_{\text{max}}$ represent the minimum and maximum pixel values of the DSA image, respectively, and ${p}_{g}$ represents the probability of pixel value $g$. The entropy quantifies the distribution of pixel values in DSA images. For DSA images with fewer artifacts, the histogram is concentrated around two points (blood vessels and background), resulting in a low entropy. By contrast, DSA images with motion artifacts exhibit a broader pixel distribution, leading to a higher entropy.

The Wilcoxon signed-rank test was employed to evaluate the significance of differences between the median scores for each patient obtained using Ohnishi’s method and the proposed method, with statistical significance defined as *p* < 0.001.

To verify the feasibility of practical clinical application, we evaluated the processing time required to generate a series of DSA images for Ohnishi’s method and the proposed method. This measurement of the processing time encompassed the entire workflow, from loading a series of mask images and a series of contrast images to generate sequential DSA images.

## Results

**Fig. 3:**
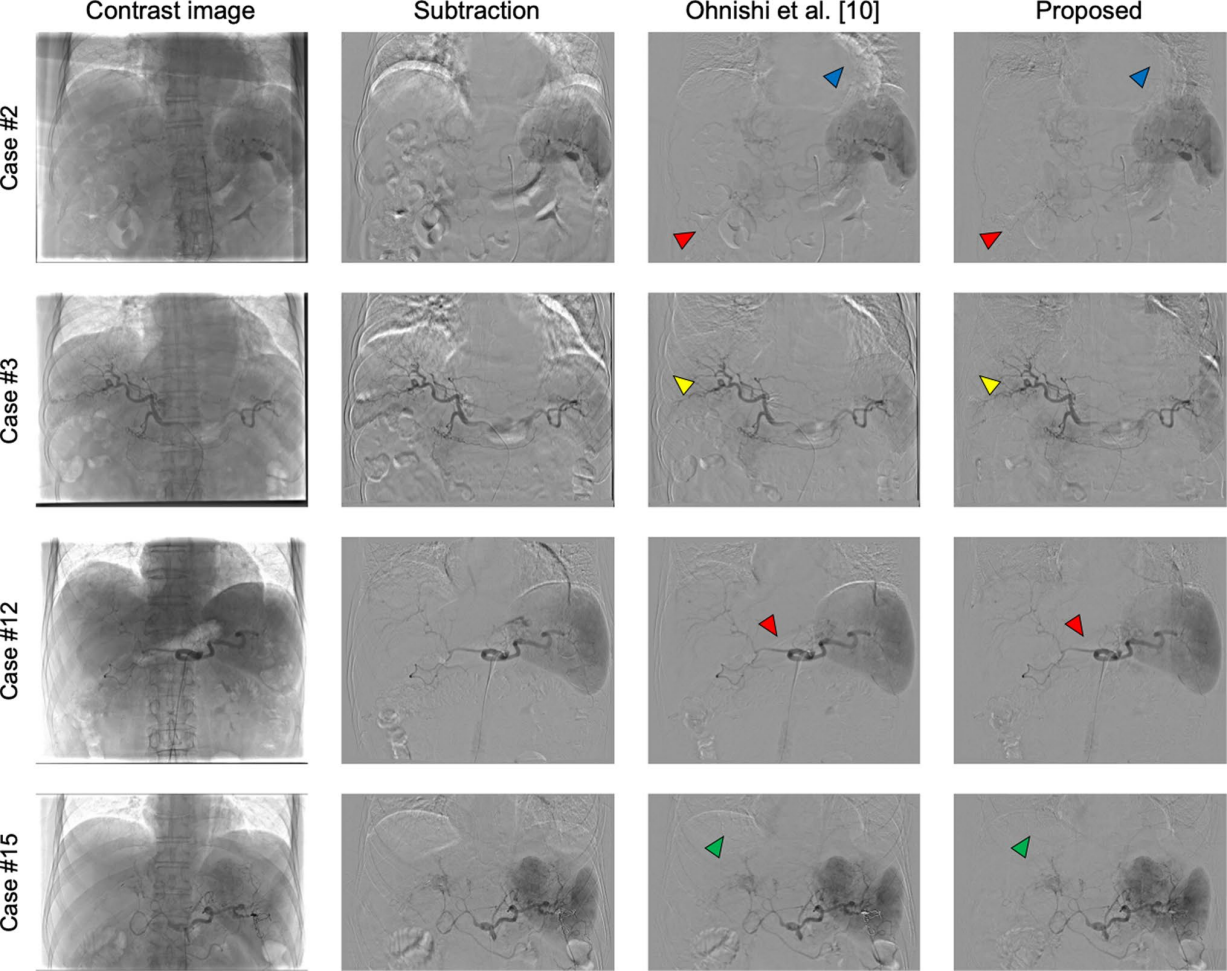
DSA images generated using different methods. From left to right, the columns correspond to contrast images, DSA images generated using simple subtraction method, DSA images generated using Ohnishi’s method, and DSA images generated using the proposed method. Red, blue, yellow, and green arrowheads indicate artifacts derived from the intestinal canals, cardiac margin, ribs, and diaphragm, respectively

Figure 3 shows a comparison of DSA images generated using the proposed method and Ohnishi’s method. We also included the results of a simple subtraction method, in which only the first image in the series of mask images was specified as the mask image for processing. Although both methods generated DSA images with few artifacts, motion artifacts caused by the misalignment of several organs were still present in the images generated using Ohnishi’s method. For example, in case #2, the proposed method effectively reduced artifacts originating from the intestinal canals and heartbeats (indicated by red and blue arrowheads, respectively), achieving clearer images relative to the comparison method. Similarly, in case #3, the proposed method reduced artifacts associated with the intestinal canals and cardiac margin, and notably reduced rib-related artifacts (yellow arrowheads). In case #12, the proposed method minimized the artifacts from the intestinal canals, thereby improving the visibility of vascular structures in the DSA images. Furthermore, in case #15, the proposed method more effectively reduced diaphragm-related artifacts (green arrowheads) relative to the comparison method.

**Fig. 4:**
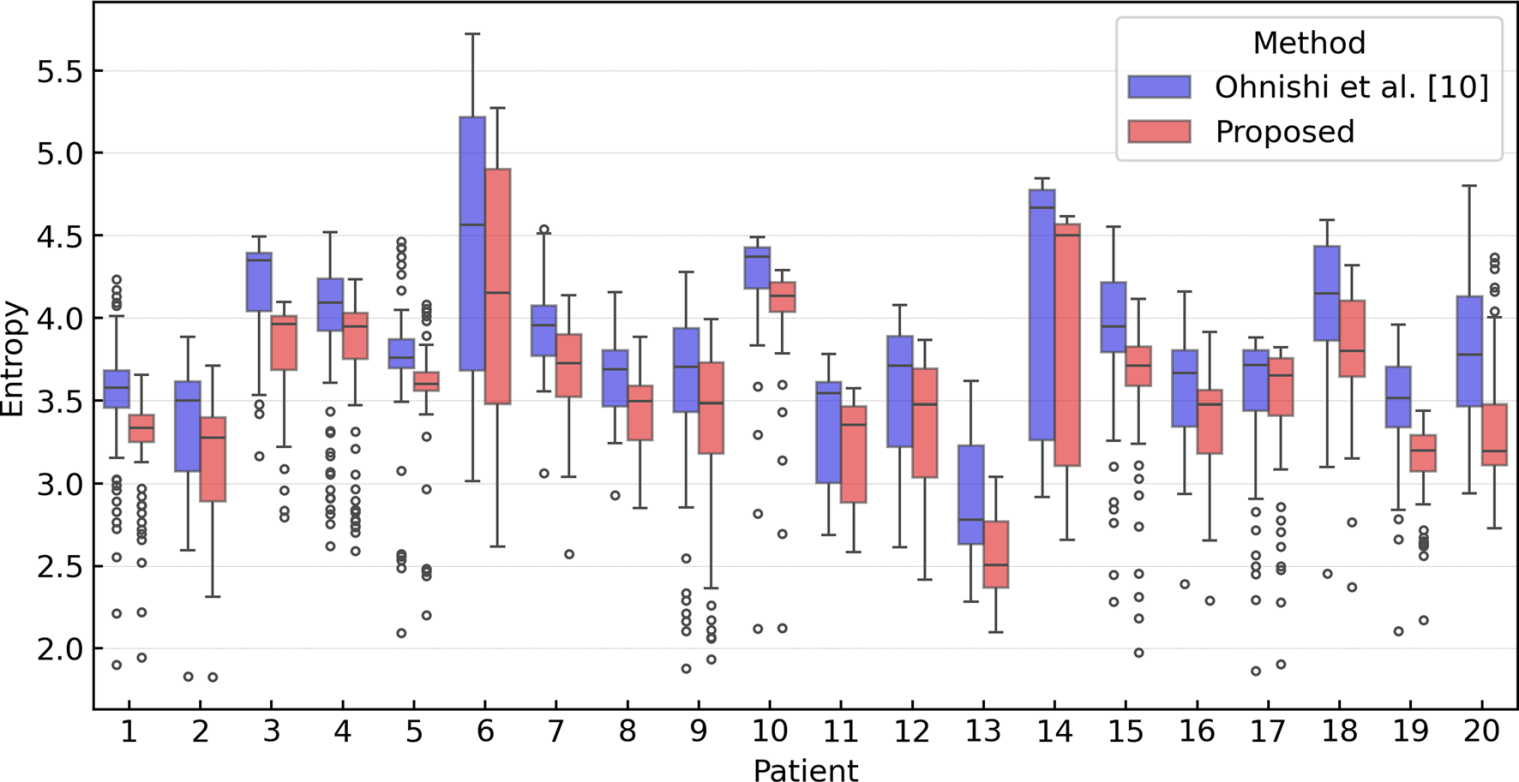
Quantitative analysis of DSA images generated using Ohnishi’s method and the proposed method with respect to entropy

Figure 4 shows boxplots of the entropy values for DSA images for each patient generated using Ohnishi's method and the proposed method. The average entropy calculated across all cases was 3.74 for Ohnishi’s method, whereas the proposed method achieved a lower value of 3.50. The proposed method significantly (*p* < 0.001) outperformed Ohnishi’s method in every case, with significant differences.

Furthermore, the average processing times across all cases for Ohnishi’s method and the proposed method were 0.116 s and 1.829 s, respectively. We confirmed that the processing time of the proposed method was longer than that of the comparison method.

## Discussion

We developed a new patch-based phase-matching approach to generate abdominal DSA images with fewer artifacts under natural breathing conditions. The proposed method leverages local information through patch-based matching instead of relying on global subtraction and applies subpixel-level refinement to improve alignment accuracy further. Since misalignment between mask and contrast images is a major cause of motion artifacts in DSA, these two key techniques can contribute to motion artifact reduction. Experimental results demonstrated that the proposed method effectively suppresses motion artifacts regardless of anatomic structure, outperforming Ohnishi’s method by achieving lower average entropy in all 20 cases. The lower entropy indicates improved uniformity in the generated DSA images. Specifically, in case #12, Ohnishi’s method eliminated artifacts in the diaphragm but not artifacts along the cardiac margin. By contrast, the proposed method successfully removed artifacts from both the diaphragm and the cardiac margin, clearly highlighting its superiority. Furthermore, we demonstrated that the proposed method completed the entire workflow within 2 s. Although the processing time was longer than that of Ohnishi’s method due to the increased number of sequential image processing steps, such as patch-based phase-matching, this duration was judged by a radiologist (JK) and three physicians (K. Fujimoto, K. Fujiwara, and TK) to be sufficiently short for practical clinical application. Overall, our results indicate that the proposed method can rapidly generate clear abdominal DSA images under natural breathing conditions within a clinically acceptable timeframe.

The dynamic observation enabled by abdominal DSA images is clinically essential, particularly in interventional treatments. For example, TAE requires accurate identification of the arteries supplying tumors, known as feeders, which is essential to embolize the arteries and suppress tumor growth. However, the abdominal environment changes dramatically during respiration, resulting in changes in blood vessel angles and leading complex vascular structures to deform and overlap on 2D images. Therefore, capturing these details with a single static image is challenging. The ability to acquire sequential DSA images enables clearer identification of feeders and is indispensable for enhancing treatment success rates. The proposed method addresses this challenge by performing detailed patch-based subtraction processing between sequential mask and contrast images, which enables the generation of high-quality sequential DSA images.

**Fig. 5:**
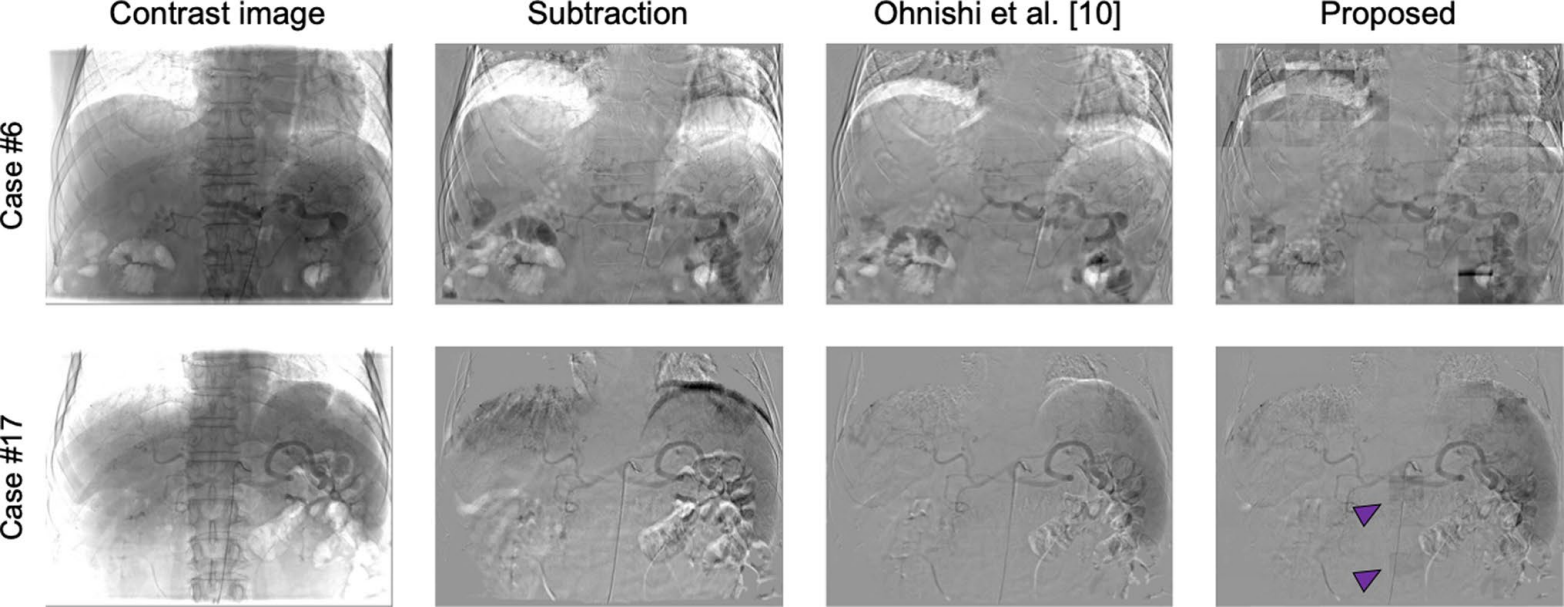
Examples of failed DSA images. From left to right, the columns correspond to contrast images, DSA images generated using simple subtraction method, DSA images generated using Ohnishi’s method, and DSA images generated using the proposed method. Purple arrowheads indicate tiling artifacts derived from inappropriate brightness correction

The proposed method was not highly effective in all cases, which exhibits some limitations of the proposed method. In case #6, for example, severe motion artifacts and tiling artifacts occurred in the DSA images generated using our method, as shown in Fig. 5. Upon examining both mask and contrast series, we confirmed that the patient’s breathing was deeper during the acquisition of contrast images than during acquisition of mask images, which hindered the image-matching process. This suggests a limitation in handling inconsistent breathing during image acquisition; however, it is a common challenge in phase-matching. The problem of insufficient images can be mitigated by instructing patients to take deep breaths during mask image acquisition. In case #17, we confirmed that inappropriate brightness correction led to tiling artifacts in some patches (purple arrowheads). The brightness correction feature of the proposed method calculates average pixel values from regions outside the contrast-enhanced areas. However, in patches in which tiling artifacts occur, although these patches do not actually contain contrast-enhanced areas, poor threshold settings can cause the thresholding process to incorrectly identify most regions as contrast-enhanced, leading the brightness correction process to generate undesirable patches. This indicates another limitation in brightness correction process due to its dependence on accurate thresholding. To improve accuracy, a more precise brightness correction method capable of accurately identifying contrast-enhanced areas should be incorporated in the future.

## Conclusion

This study proposed a patch-based phase-matching method for generating abdominal DSA images with fewer artifacts compared with the previous method under natural breathing conditions. These methods were compared using abdominal angiograms from 20 cases to evaluate the performance of the proposed method versus that of Ohnishi’s method. The proposed method generated DSA images with fewer motion artifacts and consistently achieved lower entropy values, with significant differences observed between Ohnishi’s and our methods in all cases.

## Data Availability

The data underlying this article are not publicly available due to the privacy of the individuals who participated in the study but may be shared upon reasonable request.
